# The Effects of a Skill-Based Intervention for Victims of Bullying in Brazil

**DOI:** 10.3390/ijerph13111042

**Published:** 2016-10-26

**Authors:** Jorge Luiz da Silva, Wanderlei Abadio de Oliveira, Iara Falleiros Braga, Marilurdes Silva Farias, Elisangela Aparecida da Silva Lizzi, Marlene Fagundes Carvalho Gonçalves, Beatriz Oliveira Pereira, Marta Angélica Iossi Silva

**Affiliations:** 1 College of Nursing, University of São Paulo, Ribeirão Preto, SP 14040-902, Brazil; jorgelsilva@usp.br (J.L.d.S.); wanderleio@usp.br (W.A.d.O.); iarafalleiros@usp.br (I.F.B.); malufarias@usp.br (M.S.F.); mgoncalves@eerp.usp.br (M.F.C.G.); 2 Department of Mathematics, Federal University of Technology-Paraná, Cornélio Procópio, PR 86300-000, Brazil; elisangelalizzi@utfpr.edu.br; 3 Research Centre on Child Studies, Institute of Education, University of Minho, Braga 4710-057, Portugal; beatriz@ie.uminho.pt

**Keywords:** bullying, social and emotional skills, school transition, school-based intervention

## Abstract

This study’s objective was to verify whether improved social and emotional skills would reduce victimization among Brazilian 6th grade student victims of bullying. The targets of this intervention were victimized students; a total of 78 victims participated. A cognitive-behavioral intervention based on social and emotional skills was held in eight weekly sessions. The sessions focused on civility, the ability to make friends, self-control, emotional expressiveness, empathy, assertiveness, and interpersonal problem-solving capacity. Data were analyzed through Poisson regression models with random effects. Pre- and post-analyses reveal that intervention and comparison groups presented significant reduced victimization by bullying. No significant improvement was found in regard to difficulties in practicing social skills. Victimization reduction cannot be attributed to the program. This study contributes to the incipient literature addressing anti-bullying interventions conducted in developing countries and highlights the need for approaches that do not exclusively focus on the students’ individual aspects.

## 1. Introduction

School bullying refers to acts among peers characterized by intention, repetitiveness and imbalance of power among students [1]. These acts can be physical (e.g., hitting, kicking, pushing), verbal (e.g., calling names, swearing at the victim, laughing), or relational (e.g., socially isolating the victim, spreading rumors, or manipulating relationships) [1,2]. Children and adolescents can become involved with bullying as bullies, victims, reactive-victims, or bystanders [3]. The rates at which this phenomenon occurs range among countries: between 7% and 43% refer to victims and from 5% to 44% refer to bullies [4]. Rates in Brazil range from 7% to 22% for victimization and from 17% to 21% for aggression [2,5]. The presence of bullying in the school context hinders learning and the healthy development of students [5], while also collaborating to create a perception that school is not a very safe place [6]. Bullies and victims can present higher rates of depressive symptomatology [7], anxiety [8], insecurity [9], loneliness [5], learning problems [10], juvenile delinquency [11], and suicidal ideation [12]. The negative effects of bullying indicate the need to develop interventions to prevent or reduce the occurrence of this phenomenon in schools.

The international literature shows that various anti-bullying programs have been implemented, such as whole-school anti-bullying programs [13,14], curriculum interventions [15,16], and social skills training [17,18]. A meta-analysis including 44 studies reports that the success of interventions varies. The average decrease in aggression is 23%, while victimization has been reduced by 20%, though only some programs presented significant results [19]. The most efficacious elements of interventions intended to decrease victimization include strict disciplinary methods, training for parents, meetings, videos and cooperative group work for students, as well as programs of greater duration and intensity directed to children and teachers [19]. Another two meta-analyses identified very few effects to have practical relevance [20,21].

Due to the inexpressive results presented by most anti-bullying interventions, some researchers have drawn attention to the need to implement programs that indirectly approach the phenomenon [22,23]. Indirect approaches include programs in which the prevention or reduction of bullying occur by promoting social and emotional skills and encouraging pro-social behavior that favors non-violent social interactions with peers and adults through conflict resolution and establishing friendships, for instance [24]. This type of intervention was successful in decreasing the frequency of physical aggression [25] and victimization [26] in the United States, a country where most anti-bullying programs are less successful than those implemented in European countries.

From another perspective, since only a small number of students are directly involved in bullying, the inexpressive results of most interventions may be related to the fact that they include all students [27]. Recently, a recommendation was made to implement programs exclusively directed to either bullies or victims, focusing on promoting skills among children and adolescents [28]. Lack of appropriate social skills is one of the predictors of victimization [4]. Social skills represent classes of behaviors individuals use to successfully complete a social task [29]. Bullying victims present a lack of appropriate social skills, such as social isolation and inefficient coping strategies such as crying and ignoring the bully [30]. These strategies, in general, signal to bullies that the victims lack self-defense skills, which combine with violence to become even more intensified [31].

Therefore, improved social skills, especially assertiveness, represent an important aspect upon which to ground interventions intended to reduce bullying among victims [17,18]. There are few studies addressing selective interventions directed to victims of bullying [32]. A study developed in English schools identified that the training of social skills improved the self-esteem of children, though victimization was not significantly reduced [18]. A significant reduction of victimization was reported by a study implemented in Australian schools addressing male adolescent bullying victims who presented symptoms of anxiety [33]. The components of the program developed in Australia that may have ensured its success were social skills, the objective of which was to help children establish supporting friendships and develop assertive coping strategies. The focus on emotional skills using strategies to control anger and frustration is another aspect to highlight, as it may have contributed to the intervention’s success, as well as increased the focus on anxiety [33]. In Brazil, there are no studies with this type of approach.

Internationally, the interventions with the best results are those addressing the entire school [19]. One of the lines of this approach considers bullying a group phenomenon and, for this reason, focuses on students not involved with bullying, or bystanders, because they can either defend the victim or reinforce the bullies’ behavior [14]. Having peers willing to stand up for them is very important for victims; however, victims also need to have self-defense skills and must be able to establish friendships to improve the social support they receive, which may be facilitated by the improvement of social skills. Competent social skills are more needed in some periods of school life, such as when transitioning between school cycles or levels within the organization of the educational system, so social competence is important to properly cope with changes that occur in this period [34].

Studies have shown that behavioral problems, lack of discipline and bullying become more frequent during school transitions because students have to relate to a larger number of unknown peers and make new friends and form new social groups [1,35]. A greater concern over social status during this period may encourage aggressive behavior as a way to achieve self-affirmation and become popular among peers [14,36]. A lack of appropriate social skills among victims can hinder self-defense, the establishment of friendships and social adaptation during school transitions. Hence, even though victimization tends to decrease between the ages of eight and 16 years old, a peak usually occurs in the 6th grade [37]. Such violence can negatively affect the quality of victims’ school experiences and the relationships they establish with their peers [34].

This study was developed in Brazil, a developing country in Latin America. With a pre- and post-test format, this investigation focuses on students who were victims of bullying. Mixed groups, however, were included in the intervention; that is, bystanders were also included. The reason for including bystanders is because there are indications that victims lack appropriate social skills, so gathering participants with similar difficulties into the same group may be counter-productive [38]. Hence, the intention was to allow victims to interact and make friends with non-aggressive students so that they would establish connections during the intervention and form a larger network of social support. We also expected that the bystanders would offer social support to the victims during the school routine.

This study’s objective was to verify whether improved social and emotional skills would reduce victimization among Brazilian students who were victims of bullying attending the 6th grade (first year of the equivalent to middle school in Brazil). This is the first investigation addressing the impact of an intervention based on the development of social and emotional skills on a population of Brazilian student victims of bullying. Its results can improve knowledge concerning this phenomenon in this sociocultural context and can also indicate possibilities in the design of preventive measures and the combat of bullying.

## 2. Materials and Methods

### 2.1. Participants

A total of 522 6th grade students (first year of the equivalent to middle school in Brazil) attending six schools from a Brazilian city were invited and 411 consented to participate. Among a total of 285 students assessed in the pre-test and considered to be either victims or bystanders, 203 consented to participate in the study’s second stage that involved the intervention. Thirteen boys and two girls withdrew from the study and were excluded from the final sample, which was finally composed of 188 students assigned to intervention (41.5%) and comparison (58.5%) groups.

Participants were assigned to intervention and comparison groups within their own schools. The 18 6th grade classrooms were distributed into these two conditions in order to obtain comparable samples, so that the nine classrooms composing the intervention group and the nine classrooms composing the comparison group presented similar amounts of victims, bullies and bystanders. All the victims and bystanders from the intervention group were invited to take part in the intervention and all those who agreed were included. The students were assigned to the groups according to an average proportion of 40%–50% of victims and 50%–60% of bystanders. The same occurred for sex, as there were more girls than boys. Some participants, however, withdrew from the study so that the proportion of female participants in the final sample was 72.1% in the intervention group as opposed to 58.8% in the comparison group, a difference that was not, though, statistically significant (*p* = 0.07). Altogether, 78 victims (41.5%) and 110 bystanders (58.5%) participated. From the total number of victims, 40 (51.3%) were typical victims and 38 (48.7%) were reactive-victims.

The average age in the intervention group was 11.28 years old and in the comparison group it was 11.21 years old (*p* = 0.441). The ethnic composition of the groups was similar (*p* = 0.566). The intervention group included: mixed race individuals (48.8%), Caucasians (38.4%), Afro-descendants (8.1%) and others (4.7%), while the comparison group included: mixed race participants (43.1%), Caucasians (42.2%), Afro-descendants (8.8%), and others (5.8%). Both those who participated in the survey, but not in the intervention, (*n* = 82) and bullies (*n* = 126) did not present significant differences regarding their distribution in classrooms (intervention and comparison), suggesting that those taking part in the intervention belonged to classrooms with similar characteristics.

This study’s focus was students who were victims of bullying. The participation of bystanders was an extra component in the intervention, the objective of which was to promote interaction with the victims as pro-social peers and encourage the establishment of friends (victims and bystanders) to increase the amount and quality of social support and help provided to victims. Even though the characteristics of bystanders were considered when forming the groups, only results concerning the victims are presented. The study was approved prior to implementation by the Institutional Review Board at the University of São Paulo at Ribeirão Preto, College of Nursing (Protocol CAAE: 39462414.0.0000.5393). Parents and legal guardians authorized the participation of students by signing consent forms.

### 2.2. Intervention

The students participated in a behavioral cognitive intervention based on social skills [29]. The eight weekly sessions, which lasted 50 min each, were led by a clinical psychologist (this paper’s primary author) on the schools’ premises during school hours. The groups were composed of eight to ten participants mixed by gender (female and male) and condition (victim and bystander).

The sessions addressed content and activities related to civility, the ability to make friends, empathy, self-control, and emotional expressiveness, assertiveness and interpersonal problem-solving capacity. Content and activities were developed according to guidelines established by the program [29] in order to ensure a reliable application of the intervention. The structure of the sessions was based on cognitive-behavioral techniques, such as: role-play, dramatization, positive reinforcement, modeling, feedback, videos, and homework assignments. Each meeting was organized around three points in time: (1) beginning—the participants commented on the homework assignments, received feedback, orientation and support from the group and coordinator, then a brief summary from the previous meeting was presented; (2) middle—activities programmed for the meeting were performed; (3) final—homework would be assigned and feedback on the meeting was provided by the participants and coordinator.

Homework involved practicing learned skills in different situations, real daily contexts different from that of the intervention group. Additionally, homework reports and feedback reinforced the skills learned and enabled assessing and designing new strategies if the initial attempt had not been successful. These strategies were intended to provide support to students in implementing social skills. The groups were assessed once more after the intervention (post-test).

The intervention took place between March and May 2015, at the beginning of the school year, which in Brazil starts in February and ends in December. The pre-test occurred in the first week of March and the post-test assessment took place in the first week of June (seven days after the intervention ceased) for both the intervention and comparison groups.

### 2.3. Measurements

#### 2.3.1. Self-Report (S-R)

Escala de Agressão e Vitimização entre Pares—EVAP (Aggression and Peer Victimization Scale) [39]. EVAP is an 18-item instrument that takes approximately 5 min to be completed. The participants checked the frequency with which they practiced direct or indirect aggressive behavior or were targets of such behaviors. For instance: “I pushed, punched and/or kicked other students”; “I was pushed, punched and/or kicked by other students”; “I cursed at other students”; “I was cursed at by other students”. Answers are provided on a Likert scale (1 = never; 2 = almost never; 3 = sometimes; 4 = almost always; 5 = always). Therefore, the scores for the eight questions addressing victimization ranged between 8 (minimum) and 40 (maximum) and for the questions addressing aggression, the interval ranged from 10 (minimum) to 50 (maximum). Psychometric analyses indicated good internal consistency for victimization (α = 0.81) and aggression (α = 0.79).

Sistema Multimídia de Habilidades Sociais para Crianças—SMHSC [40] (Multimedia System of Social Skills for Children, Casa do Psicólogo, São Paulo, Brazil). SMHSC is a self-assessment computerized instrument addressing social skills. It takes approximately 35 min to be completed. It is composed of 21 main videos depicting children interacting with other children and adults. Each situation presents another three short videos depicting alternative behavioral responses for the situation presented in the main video, namely: skillful, passive non-skillful, active non-skillful. The general classes of social skills that are assessed refer to empathy and civility, coping assertiveness, self-control, and participation. For instance, main video number 17 is titled “Resisting Peer Pressure” and belongs to the general class of social skill of coping assertiveness. In the video, Carlos finds out that the ball is kept in the teacher’s room and wants Bruno to go and get it, saying: “If you’re not a sissy”. The entire group confirms: “That’s right! If you’re not a sissy”. Another three videos are presented; each depicts a different response from Bruno. The first is a non-skill active response. Bruno disagrees from the boys and threat to fight saying: “I’m not a sissy! You are! Do you want a piece of me!” The second is a non-skilled passive response. Bruno agrees to do what the group demands saying: “Alright, alright, I’ll go….”. The third response is skilled. Bruno disagrees and explains: “I’m not going there just because you want! And it has nothing to do with being a sissy!” After each response, two questions are asked: 1. “Do you usually respond this way?”, to which the following options are offered: “a. always, b. sometimes, and c. never”; and 2. “What do you think about responding this way?”, to which the options are: “a. correct, b. more or less, and c. wrong”. Only for the skilled response is a third question is asked: “Do you have difficulty responding this way”, to which the options are: “a. correct, b. more or less, and c. wrong”. Answers to the question were analyzed using SMHSC, which provided raw and standardized scores for each participant. The general score for difficulty in regard to the practice of social skills was used in this study (α = 0.78).

#### 2.3.2. Peer-Report (P-R)

Sociometric scale [41]. This peer-selection instrument is composed of 10 items and its completion takes approximately 7 min. The participants indicated positive preferences of up to three classmates with whom they enjoyed hanging around, playing, talking or doing schoolwork. They also indicated the negative preference of up to three classmates with whom they least liked to hang around, play, talk or do school work. The participants also listed classmates who had the following characteristics: having few friends, being nice, and being able to resolve conflicts. All the participants who consented to participate in the study, and not only those who took part in the intervention, completed the sociometric scale. The number of indications of each sociometric item was considered within the intervention and comparison groups.

### 2.4. Statistical Analysis

First, to identify involvement in bullying based on the responses provided to the questionnaire, EVAP, grouping analyses were performed using Ward hierarchical method. The Ward method consists of a hierarchical grouping procedure in which the similarity measure used to group individuals is calculated as the sum of squares between the two groupings.

The Ward method consists of a procedure of hierarchical grouping in which the similarity measure used to gather groupings is calculated as the sum of squares between two groupings of all the variables. This method is distinct from other cluster methods because it uses an analysis of the variance approach in order to evaluate the distances between the clusters. In the Ward method, the mean distance of an observation that falls in the center of a cluster from the observations in the same cluster are taken as the basis and the total deviation squares are used. This method tends to result in groupings of approximately equal sizes due to the minimization of internal variation. Three categories emerged: 1. Bystander (low frequency of aggression and low frequency of victimization); 2. Victim (high frequency of victimization and low or moderate frequency of aggression); and 3. Bully (high frequency of aggression and low or moderate frequency of victimization). In this study, reactive-victims are those students who presented a high frequency of victimization with a moderate frequency of aggression.

Afterwards, data concerning the pre- and post-tests were described in terms of mean and standard deviation. The variables of interest had their scores compared with respect to time (pre-test and post-test) and groups (intervention and comparison) through a random effect Poisson regression [42] with log-linear canonical link function. This type of model is indicated when the response variable is a count variable. The study data do not meet normality criteria, as in the usual regression model, because the function’s domain is the real line, which is not the case for the data at hand. A random effect was included to account for the correlation arising from the fact that the same subject is observed in different periods: pre-test and post-test. Estimates of the parameters were obtained by the maximum likelihood method because the variables “few friends”, “conflict resolution” and “being nice” have standard deviations greater than their means, the model included an over dispersion [43] using SAS’s PROC GENMOD software (SAS Institute Inc., Cary, NC, USA). Throughout the analysis, a 5% level of significance was considered.

In the Poisson regression results, betas refer to orthogonal contrasts obtained for the comparisons between the variables of interest, with respect to time (pre- and post-). The orthogonal contrasts are a means of obtaining a test of a specified hypothesis concerning the model parameters. This is accomplished by specifying a matrix L for testing the hypothesis L’β = 0. The statistics calculated are based on the asymptotic chi-square distribution of the likelihood ratio statistic, for the generalized score statistic for generalized models, with degrees of freedom determined by the number of linearly independent rows in the matrix. For instance: Assume a group effect with two levels (Intervention and Comparison), to specify matrix L’. In order to test the difference between groups, we should create a matrix of one line with two columns: 1 was assigned to the first column and −1 was assigned to the second column. In this way, we would compare the groups (Intervention and Comparison) and obtain an estimated difference between the groups, estimative of difference that, in the results section of this paper is called betas. The betas presented in this paper correspond to contrast of times in inverted order, that is, post-test in regard to the pre-test having a matrix L = (−1,1). For this reason, when beta is negative, we can attribute increased acceptance to the post-intervention.

## 3. Results

Table 1 presents the differences found by the Poisson regression model for the variables with regard to the intervention and comparison groups in the pre- and post-tests.

The results indicate a significant decrease in total victimization in the intervention (β = 0.1851, SE = 0.0455, *p* < 0.0001) and comparison groups (β = 0.2617, SE = 0.0483, *p* < 0.0001). Physical victimization decreased significantly in both groups. Verbal victimization decreased significantly among the victims in the intervention (β = 0.1583, SE = 0.0674, *p* = 0.018) and comparison groups (β = 0.2742, SE = 0.0724, *p* = 0.0002). Relational victimization also decreased among victims in both the intervention (β = 0.2218, SE = 0.0777, *p* = 0.004) and comparison groups (β = 0.2409, SE = 0.0816, *p* = 0.003). No significant differences were found with regard to aggression, though total aggression decreased somewhat in the intervention group. Difficulty experienced by victims in the intervention group in terms of practicing social skills was reduced, but not significantly. Peer acceptance increased for both the intervention and comparison groups, but not significantly. The intervention group was less frequently nominated as having few friends while the comparison group was more frequently nominated as having few friends, but in both cases, the differences were not significant. Conflict resolution did not increase significantly for the victims of any of the groups. The intervention group was more frequently considered nice, though with no statistical significance.

## 4. Discussion

This study’s objective was to verify whether improved social and emotional skills would reduce victimization among Brazilian student victims of bullying attending the 6th grade (first year of the equivalent to middle school in Brazil). The results indicate a significant decrease in victimization presented by the intervention and comparison groups when comparing pre- and post-tests. Similar results in the intervention group are reported by another study that implemented interventions involving social and emotional skills [33]. In this study, however, a significant decrease in victimization was also presented by the comparison group, thus we cannot claim that the positive results observed in the intervention group were due to the program developed in this study addressing social and emotional skills. A potential explanation involves difficulties faced by students at the beginning of the 6th grade, such as changing schools, the need to interact with new students and adapt to significant changes in school structure, disciplinary control and expectations concerning academic performance [34]. For this reason, victimization rates are possibly higher at the beginning of the year when students were assessed in the pre-test and these initial difficulties concerning peer interactions, conflicts, violent situations and bullying may have decreased as students came to feel better adapted to the new school situation and after having made new friends, the situation that was reflected in the post-test.

Even though it was not statistically significant, aggression decreased in the intervention group. This result may be related to improved social skills, as this indicates a tendency of the victims in the intervention group to act with more civility, empathy, and self-emotional control, and to solve related problems in a non-violent way. This is important because, even though victims generally respond to aggression in a passive manner (exhibiting submission or crying easily, for instance), which may reinforce aggressiveness because a passive response signals to bullies that their actions are successful [31], an aggressive response may also increase the frequency with which intimidating situations occur over time [44]. The short-term interaction between victims and bystanders during the sessions was not sufficient to significantly increase the network of peers among those participating in the intervention; the post-test still indicated the participants had few friends. The short period between the pre-test and post-test may have also influenced the results due to “social status rigidity”, which requires time to be changed in social relationships [18]. Having the support of peers when facing bullying is important because even the most assertive responses of victims in the face of aggression may be ineffective in a context in which bullies have high social status or aggression is considered to be normal [36].

This study presents some limitations. First, the post-test was implemented one week after the intervention. A longer period of time would be more suitable to assess changes in bullying and social skills. Another limitation was that the activities were performed within an “artificial environment”; the context of a classroom provides more opportunities to intervene in real daily situations and test learned skills, although there was an effort in this study to make the intervention environment as close as possible to that which the participants routinely experience in their interactions. Further studies should overcome these limitations and incorporate the intervention developed in this study into the school curriculum so that this would be a role to be performed by teachers. Waiting a longer period between the pre-test and post-test is also recommended. The instrument used to collect data regarding bullying does not address cyber bullying, which represents another limitation in this study.

## 5. Conclusions

The intervention group experienced less difficulty to a statistically non-significant degree with regard to social skills. Victimization decreased significantly in both the intervention and comparison groups. Aggressiveness did not significantly decrease. This is the first study testing an intervention based on social and emotional skills directed at victims of bullying in Brazil, with the potential to encourage reflection upon intervention models to be designed for the Brazilian context.

## Figures and Tables

**Table 1:** Comparison between groups (intervention and comparison) with regard to pre-test and post-test using Poisson regression model.

Measure	Intervention (*n* = 38)		Comparison (*n* = 40)	
	Pre-Test	Post-Test	Pre-Test	Post-Test
S-R Total Victimization	26.63 (4.92)	22.13 (7.27) **	25.95 (4.27)	19.97 (8.79) **
S-R Physical Victimization	5.38 (2.23)	4.48 (2.14)	5.34 (2.26)	4.55 (2.20)
S-R Verbal Victimization	11.95 (2.33)	10.20 (3.35) *	11.63 (2.02)	8.84 (3.79) **
S-R Relational Victimization	9.30 (2.40)	7.45 (3.30) **	8.97 (2.57)	7.05 (3.16) **
S-R Total Aggression	20.50 (5.38)	19.85 (5.90)	19.89 (6.71)	20.03 (6.75)
S-R Physical Aggression	5.23 (2.02)	5.18 (2.02)	5.55 (2.37)	5.76 (2.27)
S-R Verbal Aggression	7.15 (2.26)	7.10 (2.65)	7.24 (2.87)	6.95 (2.67)
S-R Relational Aggression	8.18 (2.91)	7.83 (2.63)	7.11 (2.81)	7.32 (2.92)
S-R Difficulty with social skills	1.95 (1.06)	1.58 (1.14)	1.26 (0.92)	1.26 (1.21)
P-R Acceptance	4.83 (3.57)	5.33 (3.14)	3.74 (2.89)	4.58 (2.98)
P-R Few friends	1.03 (1.39)	0.93 (1.80)	0.32 (0.53)	0.47 (0.83)
P-R Conflict resolution	0.48 (0.85)	0.68 (1.35)	0.47 (0.80)	0.68 (1.69)
P-R Being nice	0.90 (1.37)	1.15 (1.76)	0.55 (0.72)	0.53 (0.86)

Notes: P-R = peer-report. S-R = self-report. Data presented as mean (standard deviation). * = *p* < 0.05, ** = *p* < 0.01.

## References

[1] Olweus D. (2013). School bullying: Development and some important challenges. Annu. Rev. Clin. Psychol..

[2] Sampaio J.M.C., Santos G.V., Oliveira W.A., Silva J.L., Medeiros M., Silva M.A.I. (2015). Emotions of students involved in cases of bullying. Texto Contexto Enferm..

[3] Silva J.L., Oliveira W.A., Sampaio J.M.C., Farias M.S., Alencastro L.C.S., Silva M.A.I. (2015). How do you feel? Students’ emotions after practicing bullying. Rev. Eletronica Enferm..

[4] Cook C., Williams K., Guerra N., Kim T., Jimerson S., Swearer S., Espelage D. (2010). Variability in the prevalence of bullying and victimization: A cross-national and methodological analysis. Handbook of Bullying in Schools: An International Perspective.

[5] Oliveira W.A., Silva M.A.I., Silva J.L., Mello F.C.M., Prado R.R., Malta D.C. (2016). Associations between the practice of bullying and individual and contextual variables from the aggressors’ perspective. J. Pediatr..

[6] Skrzypiec G., Slee P., Murray-Harvey R., Pereira B. (2011). School bullying by one or more ways: Does it matter and how do students cope?. Sch. Psychol. Int..

[7] Williford A., Boulton A.J., Jenson J.M. (2014). Transitions between subclasses of bullying and victimization when entering middle school. Aggress. Behav..

[8] Bauman S., Del Rio A. (2006). Preservice teachers’ responses to bullying scenarios: Comparing physical, verbal, and relational bullying. J. Educ. Psychol..

[9] Sampson R. (2009). Bullying in Schools.

[10] Duy B. (2013). Teachers’ attitudes toward different types of bullying and victimization in Turkey. Psychol. Sch..

[11] Silva J.L., Bazon M.R. (2015). Education and juvenile delinquency: An integrative literature review. Estud. Psicol. (Natal).

[12] Kim Y.S., Leventhal B.L., Koh Y.J., Boyce W.T. (2009). Bullying increased suicide risk: Prospective study of Korean adolescents. Arch. Suicide Res..

[13] Wurf G. (2012). High school anti-bullying interventions: An evaluation of curriculum approaches and the method of shared concern in four Hong Kong international schools. Aust. J. Guid. Couns..

[14] Yang A., Salmivalli C. (2015). Effectiveness of the KiVa antibullying programme on bully-victims, bullies and victims. Educ. Res..

[15] Rawana J., Norwood S., Whitley J. (2011). A mixed-method evaluation of a strength-based bullying prevention program. Can. J. Sch. Psychol..

[16] Stevens V., Bourdeaudhuij I., Van Oost P. (2000). Bullying in Flemish schools: An evaluation of anti-bullying intervention in primary and secondary schools. Br. J. Educ. Psychol..

[17] DeRosier M.E. (2004). Building relationships and combating bullying: Effectiveness of a school-based social skills group intervention. J. Clin. Child Adolesc. Psychol..

[18] Fox C.L., Boulton M.J. (2003). Evaluating the effectiveness of a social skills training (SST) programme for victims of bullying. Educ. Res..

[19] Ttofi M.M., Farrington D.P. (2011). Effectiveness of school-based programs to reduce bullying: A systematic and meta-analytic review. J. Exp. Criminol..

[20] Ferguson C.J., San Miguel C., Kilburn J.C., Sanchez P. (2007). The effectiveness of school-based anti-bullying programs: A meta-analytic review. Crim. Justice Policy Rev..

[21] Merrell K.W., Gueldner B.A., Ross S.W., Isava D.M. (2008). How effective are school bullying intervention programs? A meta-analysis of intervention research. Sch. Psychol. Quart..

[22] Salmivalli C. (1999). Participant role approach to school bullying: Implications for intervention. J. Adolesc..

[23] Elledge L.C., Cavell T.A., Ogle N.T., Newgent R.A. (2010). School-based mentoring as selective prevention for bullied children: A preliminary test. J. Prim. Prev..

[24] Wolfe D.A., Crooks C.V., Chiodo D., Hughes R., Ellis W. (2012). Observations of adolescent peer resistance skills following a classroom-based healthy relationship program: A post-intervention comparison. Prev. Sci..

[25] Espelage D.L., Low S., Polanin J.R., Brown E.C. (2013). The impact of a middle school program to reduce aggression, victimization, and sexual violence. J. Adolesc. Health.

[26] Jenson J.M., Dieterich W.A. (2007). Effects of a skills-based prevention program on bullying and bully victimization among elementary school children. Prev. Sci..

[27] Swearer S.M., Espelage D.L., Vaillancourt T., Hymel S. (2010). What can be done about school bullying? Linking research to educational practice. Educ. Res..

[28] Ttofi M.M., Farrington D.P., Losel F. (2012). School bullying as a predictor of violence later in life: A systematic review and meta-analysis of prospective longitudinal studies. Aggress. Violent Behav..

[29] Del Prette Z.A.P., del Prette A. (2013). Psicologia das Habilidades Sociais na Infância: Teoria e Prática.

[30] Fox C.L., Boulton M.J. (2005). The social skills problems of victims of bullying: Self, peer and teacher perceptions. Br. J. Educ. Psychol..

[31] Crawford A.M., Manassis K. (2011). Anxiety, social skills, friendship quality, and peer victimization: An integrated model. J. Anxiety Disord..

[32] Elledge L.C., Cavell T.A., Ogle N.T., Malcolm K.T., Newgent R.A., Faith M.A. (2010). History of peer victimization and children’s response to school bullying. Sch. Psychol. Q..

[33] Berry K., Hunt C.J. (2009). Evaluation of an intervention program for anxious adolescent boys who are bullied at school. J. Adolesc. Health.

[34] Silva J.L., Oliveira W.A., Bazon M.R., Cecilio S. (2013). Bullying in the classroom: Teachers’ perception and intervention. Arq. Bras. Psicol..

[35] Farmer T.W., Xie H. (2007). Aggression and school social dynamics: The good, the bad, and the ordinary. J. Sch. Psychol..

[36] Champion K., Vernberg E., Shipman K. (2003). Nonbullying victims of bullies: Aggression, social skills, and friendship characteristics. J. Appl. Dev. Psychol..

[37] Horne A.M., Stoddard J.L., Bell C.D. (2007). Group approaches to reducing aggression and bullying in school. Group Dyn.-Theor. Res..

[38] Leadbeater B., Hoglund W. (2006). Changing the social contexts of peer victimization. J. Can. Acad. Child Adolesc. Psychiatry.

[39] Cunha J.M., Weber L.N.D., Steiner Neto P., Weber L.N.D., Dessen M.A. (2009). Escala de Vitimização e Agressão entre Pares (EVAP). Pesquisando a Família—Instrumentos Para Coleta e Análise de Dados.

[40] Del Prette A., del Prette Z.A.P. (2005). Sistema Multimídia de Habilidades Sociais de Crianças.

[41] Martins M.J.D. (2009). Maus Tratos Entre Adolescentes na Escola.

[42] Cameron C., Trivedi P.K. (2013). Regression Analysis of Count Data.

[43] McCullagh P., Nelder J.A. (1989). Generalized Linear Models.

[44] Sentse M., Kretschmer T., Salmivalli C. (2015). The longitudinal interplay between bullying, victimization, and social status: Age-related and gender differences. Soc. Dev..

